# Direct Growth of AlGaN Nanorod LEDs on Graphene-Covered Si

**DOI:** 10.3390/ma11122372

**Published:** 2018-11-26

**Authors:** Fang Ren, Yue Yin, Yunyu Wang, Zhiqiang Liu, Meng Liang, Haiyan Ou, Jinping Ao, Tongbo Wei, Jianchang Yan, Guodong Yuan, Xiaoyan Yi, Junxi Wang, Jinmin Li, Dheeraj Dasa, Helge Weman

**Affiliations:** 1 Research and Development Center for Solid State Lighting, Institute of Semiconductors, Chinese Academy of Sciences, Beijing 100083, China; rf@semi.ac.cn (F.R.); yinyue@semi.ac.cn (Y.Y.); wyyu@semi.ac.cn (Y.W.); liangmeng@semi.ac.cn (M.L.); tbwei@semi.ac.cn (T.W.); yanjc@semi.ac.cn (J.Y.); jxwang@red.semi.ac.cn (J.W.); jmli@red.semi.ac.cn (J.L.); 2 Center of Materials Science and Optoelectronics Engineering, University of Chinese Academy of Sciences, Beijing 100049, China; 3 Beijing Engineering Research Center for the 3rd Generation Semiconductor Materials and Application, Beijing 100083, China; 4 Department of Photonics Engineering, Technical University of Denmark, Ørsteds Plads 345A, DK-2800 Kongens Lyngby, Denmark; haou@fotonik.dtu.dk; 5 Department of Electrical and Electronic Engineering, The University of Tokushima, 2-1, Minami-josanjima, Tokushima 770-8506, Japan; jpao@xidian.edu.cn; 6 CrayoNano AS, Sluppenvegen 6, NO-7037 Trondheim, Norway; dheeraj.dasa@crayonano.com; 7 Department of Electronics and Telecommunications, Norwegian University of Science and Technology (NTNU), NO-7491 Trondheim, Norway

**Keywords:** AlGaN, nanorod LEDs, graphene, MOCVD

## Abstract

High density of defects and stress owing to the lattice and thermal mismatch between nitride materials and heterogeneous substrates have always been important problems and limit the development of nitride materials. In this paper, AlGaN light-emitting diodes (LEDs) were grown directly on a single-layer graphene-covered Si (111) substrate by metal organic chemical vapor deposition (MOCVD) without a metal catalyst. The nanorods was nucleated by AlGaN nucleation islands with a 35% Al composition, and included n-AlGaN, 6 period of AlGaN multiple quantum wells (MQWs), and p-AlGaN. Scanning electron microscopy (SEM) and electron backscatter diffraction (EBSD) showed that the nanorods were vertically aligned and had an accordant orientation along the [0001] direction. The structure of AlGaN nanorod LEDs was investigated by scanning transmission electron microscopy (STEM). Raman measurements of graphene before and after MOCVD growth revealed the graphene could withstand the high temperature and ammonia atmosphere in MOCVD. Photoluminescence (PL) and cathodoluminescence (CL) characterized an emission at ~325 nm and demonstrated the low defects density in AlGaN nanorod LEDs.

## 1. Introduction

GaN-based semiconductor materials including AlGaN and InGaN have been considered as ideal materials for light emitting diodes (LED), laser diodes (LD), solar cells, and other optoelectronic devices on account of their direct bandgap characteristics and adjustable bandgap width. However, due to the lack of large-scale readily available single crystal substrate, GaN-based materials are usually heteroepitaxial by metal organic chemical vapor deposition (MOCVD) or molecular beam epitaxy (MBE) on sapphire, Si, or SiC, which have large lattice and thermal expansion mismatch.

To avoid defects such as dislocations or stacking faults caused by strain during the epitaxial process, a novel approach is proposed involving the growth of nanostructures such as nanorods, which have a high aspect-ratio and large surface-to-volume ratio, consequently releasing the strain and reducing the dislocation density in the upper part of the nanorods [1,2]. Moreover, core/shell nanorod structures are reported to be beneficial to increase the overall area of emission via the regrowth of the active region shell on the nanorod core, resulting in the improvement of total light intensity of the same substrate area [3], as well as avoiding the strong spontaneous and piezoelectric polarization fields through the high quality m-plane nonpolar facets [4]. Coulon et al. [5,6] reported the fabrication of core-shell LED structures using an original hybrid top-down/bottom-up approach, which achieved emission at the deep ultraviolet band. Zhuang et al. [7] developed a soft UV-curing nanoimprint lithography (NIL) technique for fabricating GaN nanogratings and nanorods, followed by reactive ion etching (RIE) and inductively coupled plasma (ICP) system processes. Our group also reported the controlled growth of GaN nanowires by a metal-catalyzed method using hydride vapor phase epitaxy (HVPE) system and achieved the orientation-controllable GaN nanowires with a high aspect ratio and excellent crystal quality [8,9].

However, the above methods for nanorods needed an original AlN or GaN template or metal catalyst—which complicated the fabrication of nanostructures. Graphene, a two-dimensional planar configuration of sp^2^-bonded carbon atoms, has attracted a great interest owing to the hexagonal arrangement of C atoms, making the one-atomic layer graphene able to serve as a nearly lattice-matched buffer for the growth of wurtzite GaN. The GaN-based nanorods can be directly grown on graphene-covered substrates without the need of a crystalline bulk or metal catalyst. Besides, graphene has other excellent physical and chemical properties, such as high optical transparency, low electrical resistivity, and mechanical strength and flexibility [10,11,12]. Furthermore, graphene films are transferable to almost any carrier substrate, including amorphous and flexible materials [13]. Therefore, the growth of GaN-based nanorods on a graphene buffer provides a new idea for fabrication of flexible optoelectrical devices. Chung et al. [14] reported the fabrication of bendable LED using high-quality GaN microdisks grown on patterned graphene microdots by epitaxial lateral overgrowth (ELOG). Heilmann et al. [15] demonstrated c-axis-oriented growth of vertically aligned GaN nanorods using single-layer graphene as an atomically thin buffer layer. Kumaresan et al. [13] reported epitaxial growth of defect-free GaN nanowires on graphene using molecular beam epitaxy without any catalyst or intermediate layer. In our previous work, we studied the direct growth of high-quality AlN films on graphene buffer, and the XRD showed the full width at half maximum (FWHM) values for (0002) and ($10\bar{1}2$) reflections were 360 and 622.2 arcsec, respectively, which were lower than that of the film directly grown on sapphire [16,17]. Moreover, we also demonstrated the GaN-based LEDs grown on multilayer graphene, which showed a higher output power than those grown on conventional sapphire [10].

In this work, we demonstrated a self-organized growth of AlGaN nanorods with a full LED structure on single-layer graphene-covered Si substrate without a metal catalyst by MOCVD. The nanorods was nucleated by AlGaN nucleation islands with a high Al composition, and grew vertically with [0001] orientation. The morphology, orientation, crystal structure, and optical properties were analyzed.

## 2. Materials and Methods

**Figure 1:**
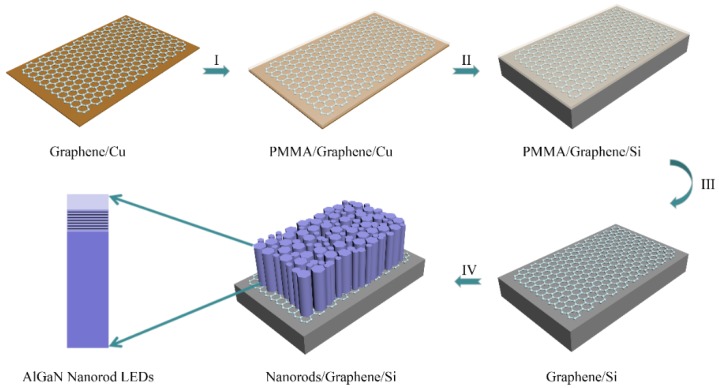
Schematic diagram of the key processes involved in the graphene transfer procedure and the metal organic chemical vapor deposition (MOCVD) growth of AlGaN nanorod LEDs: (**I**) Spin-coated poly(methyl methacrylate) (PMMA) onto graphene on Cu foil; (**II**) transfer of graphene with PMMA onto Si substrate; (**III**) dissolving PMMA; and (**IV**) MOCVD growth of AlGaN nanorod light emitting diodes (LEDs).

The key processes involved in the graphene wet-transfer procedure and the MOCVD growth of AlGaN nanorods are schematically shown in Figure 1. Prior to MOCVD growth of AlGaN nanorod LEDs, the single-layer graphene film grown on Cu foil by atmospheric-pressure chemical vapor deposition (APCVD, Xicheng, Xiamen, China) was transferred onto a Si (111) substrate. To ensure the high quality of graphene film, poly(methyl methacrylate) (PMMA) was spin-coated onto the Cu foil and baked at 120 °C for 15 min. Then, the Cu foil was immersed into an aqueous solution of iron trichloride (FeCl_3_) for 4 h to dissolve the Cu substrate entirely. After that, the graphene with PMMA needed to be transferred into deionized water two or three times to wash away residual FeCl_3_. Then, the graphene was transferred onto cleaned Si (111) substrate and dried in nitrogen. Finally, PMMA was removed using acetone and ethanol.

Throughout the growth process, we adopted trimethylgallium (TMGa), trimethylaluminum (TMAl), and ammonia (NH_3_) as precursors, silane (SiH_4_) and magnesocene (Cp_2_Mg) as dopants, and hydrogen (H_2_) as the carrier gas. The gas flow rate of precursors and dopants for all the MOCVD processes was summarized in Table 1. Before growth initiation, a nitridation step was utilized by introducing NH_3_ with a flow of 1000 sccm at 1090 °C for 5 min. Due to the higher adsorption energy and lower migration energy barrier on graphene than Ga atom, the use of Al atoms was beneficial for the adsorption at the growth interface without the participant of defects and facilitated the formation of nucleation points which supported the growth of nanorods [18]. We grew n-AlGaN nucleation islands with a relatively high Al component of 35% for 42 s by introducing TMGa and TMAl with flux of 17.5 and 30 sccm, while the flux of NH_3_ was 1000 sccm. Subsequently, the n-AlGaN nanorods with an Al component of 11% were grown at the same temperature for 25 min by introducing SiH_4_ into the reactor. The flow of TMGa and TMAl are 35 and 290 sccm respectively. Lin et al. [19,20] demonstrated that a lower V/III molar ratio could increase the vertical-to-lateral aspect ratio, consequently promoting vertical growth of nanorods. Therefore, the NH_3_ flow was 15 sccm during the whole process of nanorods growth. The MQWs structure contained 6 pairs of undoped Al_0.04_Ga_0.96_N/Al_0.11_Ga_0.89_N, and both the growth time of walls and barriers were 1 min. Finally, a thin Mg doped p-AlGaN layer was grown for 6 min.

The morphology, orientation, and crystal structure of the AlGaN nanorod LEDs were characterized by scanning electron microscopy (SEM, Hitachi, Tokyo, Japan), electron backscatter diffraction (EBSD, Zeiss, Jena, Germany) and scanning transmission electron microscopy (STEM, Tecnai, Hillsboro, OR, USA), respectively. The Raman spectra (Horiba, Kyoto, Japan) of graphene on Si substrate before and after MOCVD growth were collected using a 532-nm laser, which was excited using an argon ion laser. At last, the optical properties including temperature dependent photoluminescence (PL) and cathodoluminescence (CL) mappings of nanorods were analyzed.

## 3. Results and Discussion

**Figure 2:**
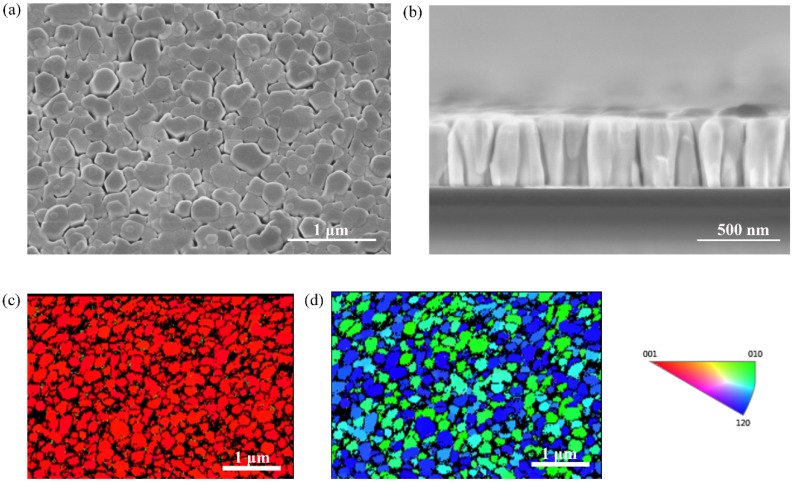
The morphology and orientation of AlGaN nanorod LEDs: (**a**) The 25° tilted-view SEM image of AlGaN nanorod LEDs; (**b**) the cross-sectional SEM image of AlGaN nanorod LEDs; (**c**) the normal-direction electron backscatter diffraction (EBSD) inverse pole figure (IPF) image of AlGaN nanorod LEDs; (**d**) the transverse-direction EBSD IPF image of AlGaN nanorod LEDs.

The morphology of AlGaN nanorod LEDs grown on the graphene was investigated by scanning electron microscopy (SEM). Figure 2a,b showed the 25° tilted-view SEM image and cross-sectional SEM image of the AlGaN nanorod LEDs grown on graphene. The nanorods were vertically aligned and had a uniform height of 440 ± 10 nm and diameter of 200 ± 10 nm. In order to investigate the grown orientation of nanorods, we further obtained the electron backscatter diffraction (EBSD) inverse pole figure (IPF). In the wurtzite nitride materials, the (0001) plane usually referred to a plane composed of alternating diatomic close packed of Ga/Al and N pairs, and the [0001] direction, also called c-axis orientation, was perpendicular to the (0001) plane. In term of MOCVD, nitrides materials were usually grown along c-axis orientation. Figure 2c showed the normal-direction IPF image of AlGaN nanorods. It was obvious that almost all AlGaN nanorods exhibited red color in the normal-direction, which demonstrated that the AlGaN nanorods had an accordant orientation along the c-axis direction. Nevertheless, blue and green colors were observed in the transverse-direction IPF image, as shown in Figure 2d. The variations of the inplane orientation of AlGaN nanorods may be due to the random growth of AlGaN nucleation islands on the graphene with different orientation.

**Figure 3:**
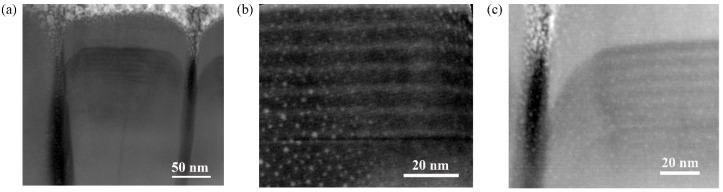
The structure of AlGaN nanorod LEDs: (**a**) cross-sectional STEM image of a single AlGaN nanorod LED; (**b**) the amplified MQWs structure; and (**c**) the curved downward MQWs and p-AlGaN.

We further investigated the structural characteristics of the GaN nanorod LEDs by STEM using a high angle annular dark field (HAADF) detector. The STEM acceleration voltage was set to 300 kV to analyze the nanostructure. Some bright spots could be observed in Figure 3, especially in Figure 3b, which were metal precipitations introduced during the sample preparation process by Focused Ion Beam (FIB). Figure 3a showed the cross-sectional STEM image of a single AlGaN nanorod LED. From bottom to top, three parts could be observed obviously including n-AlGaN region, MQWs active region, and p-AlGaN layer. Figure 3b showed the amplified MQWs structures. Six pairs of Al_0.04_Ga_0.96_N/Al_0.11_Ga_0.89_N MQWs with a uniform thickness of 8 nm could be observed with abrupt interfaces. At the top edge of nanorods, as shown in Figure 3c, the MQWs and p-AlGaN was curving downward, which was like a core-shell structure. However, due to a high density of AlGaN nanorods, the core-shell structure was not formed completely. 

**Figure 4:**
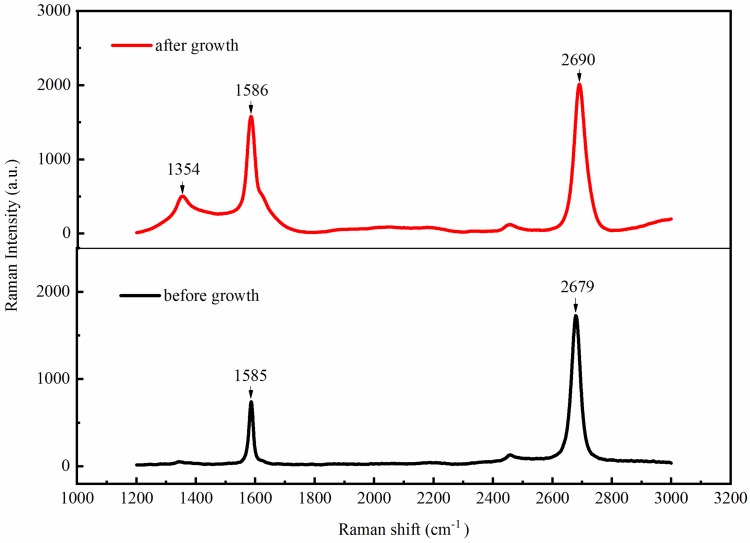
The Raman spectra of graphene’s responses before and after MOCVD growth.

Figure 4 summarized the Raman spectra of graphene’s responses before and after MOCVD growth. The G and 2D peaks (~1580 and ~2700 cm^−1^, respectively) could be observed clearly in both spectra, which demonstrated that graphene could withstand the severe growth conditions for AlGaN nanorod LEDs in MOCVD. However, compared with the spectrum before MOCVD growth, the D peak, at 1354 cm^−1^, was visible clearly after growth, illustrating the formation of defects during the process of growth on account of the etching by ammonia. These defects could increase the resistivity of grapheme [21]. Furthermore, before the MOCVD growth, the line-shape of 2D peak was systematical and the ratio of the intensity of the 2D peak to the G peak was about 2.3, which revealed the characteristic for single-layer grapheme [22,23]. After MOCVD growth, the G and 2D peaks both shifted to higher frequencies, attributed to nitrogen doping of graphene and the compressive strain during MOCVD growth, especially in the nitridation step [24].

**Figure 5:**
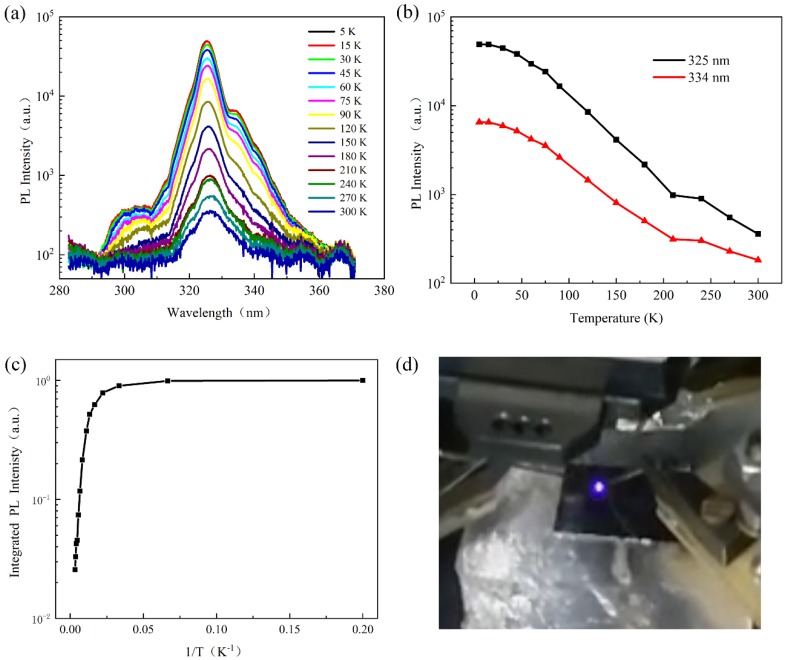
The photoluminescence properties and electroluminescence of AlGaN nanorod LEDs: (**a**) The spectra of temperature dependent PL; (**b**) the temperature dependence of PL intensity of peaks at 325 and 334 nm; (**c**) the temperature dependence of normalized integrated PL intensity of the AlGaN nanorod LEDs; and (**d**) optical image of the violet electroluminescence from the AlGaN nanorod LEDs.

We measured the photoluminescence (PL) spectra of AlGaN nanorod LEDs from 5 to 300 K using a mode-locked Ti: sapphire laser (Coherent Mira 900, Santa Clara, CA, USA) with a wavelength of 800 nm as the optical excitation source. A third harmonic generator (THG, APE, Berlin, Germany) was used to excite the sample by an output wavelength of 267 nm. Laser power incident on the sample was kept below 1 mW and the sample was cooled by liquid nitrogen. From Figure 5a, it was obvious that with the increasing of the temperature, the PL peak at 325 nm, corresponding to the emission from AlGaN MQWs active region, did not significantly shift. The full width half maximum (FWHM) of the PL peak at 300 K was 11.57 nm. On the other hand, another minor peak at 334 nm could be observed at low temperature, possibly attributed to donor–acceptor pair (DAP) emission [25,26]. Figure 5b showed the temperature dependence of PL intensity of peaks at 325 and 334 nm. PL intensity had an apparent decrease with the increasing of the temperature, which was a characteristic behavior related to a thermally activated process. Figure 5c showed the normalized integrated PL intensity as a function of temperature. To estimate the internal quantum efficiency (IQE), most of the authors usually measured PL at a certain excitation condition and assume PL internal quantum efficiency at low temperature was equal to 100% regardless of nonradiative recombination [27]. The IQE could be approximately estimated by I_PL_(RT)/I_PL_(LT), wherein I_PL_(RT) and I_PL_(LT) were the integrated PL intensity measured at room temperature (300 K) and low temperature (5 K), respectively [28,29]. The internal quantum efficiency of our sample at room temperature was calculated as 2.6%. Liu et al. [30] considered that except for the excitation power, the internal quantum efficiency of nanowire/nanorod devices may also be affected by the presence of surface states/defects, and the large bandgap AlGaN shell coverage could improve the IQE by reducing the effect of surface recombination on the quantum efficiency. On the other hand, the MQWs structure and the growth parameters in our samples needed to be optimized to further improve the IQE. Furthermore, we investigated the electroluminescence of AlGaN nanorod LEDs at room temperature. As shown in Figure 5d, the AlGaN nanorod LEDs emitted violet electroluminescence at 50 mA.

**Figure 6:**
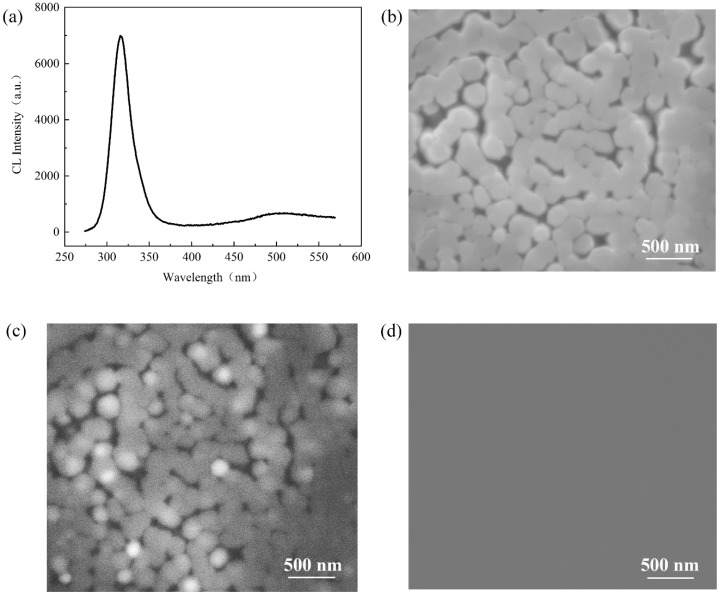
The cathodoluminescence properties of AlGaN nanorod LEDs: (**a**) The CL spectrum at room temperature; (**b**) the SEM image of CL mapping; (**c**) the CL mapping image at the wavelength of 318 nm; and (**d**) the CL mapping image at the wavelength of 500 nm.

To give a further insight of the crystalline quality of our nitride nanorods, the cathodoluminescence (CL) properties were investigated with an electron beam acceleration voltage of 15 kV at room temperature. Figure 6a shows the CL spectrum measured from the region in Figure 6b. Like PL, the CL peak at approximately 318 nm, from AlGaN MQWs active region, was clearly observed. The FWHM of the CL peak was 26.86 nm. From the CL mapping image at the wavelength of 318 nm, as shown in Figure 6c, almost all the AlGaN nanorods showed clear luminescence. In particular, strong luminescence was observed from some isolated nanorods, which had a hexagonal c-axis-oriented shape. It was suggested that these nanorods had a higher crystallinity and lower density of grain boundary than those coalesced. The coalescence of nanorods was mainly considered from two aspects. One was the density of the AlGaN nucleation islands, which determined the density of the nanorods. The density of nucleation islands was mainly affected by Al composition; a higher Al composition would lead to a higher nucleation density. Another was the lateral growth rate of the nanorods, which contributed to the coalescence between the nanorods. Lateral growth was mainly affected by temperature, pressure and V/III ratio. For example, a low V/III ratio promoted the vertical growth of the nanorods. The mechanism of the coalescence in nanorods growth required more research to further improve the controllability and crystal quality of nanorods. Furthermore, a weak yellow luminescence band could be observed in Figure 6a. To research the yellow luminescence band, the CL mapping was measured at the wavelength of 500 nm, as shown in Figure 6d. There was no detective emitting luminescence at 500 nm, which showed the yellow luminescence band was negligible and revealed the high quality and low defects density of AlGaN nanorod LEDs.

## 4. Conclusions

In summary, we achieved the direct growth of AlGaN nanorod LEDs on single-layer graphene by metal organic chemical vapor deposition (MOCVD) without a metal catalyst. The nanorods were nucleated by AlGaN nucleation islands with a high Al composition, and included n-AlGaN, 6 period of AlGaN MQWs and p-AlGaN. The morphology, orientation, and crystal structure of the nanorods were characterized to show the uniform height and diameter as well as accordant orientation along the c-axis direction. Photoluminescence (PL) and cathodoluminescence (CL) were investigated to demonstrate the high optical properties and low defects density of AlGaN nanorods. The electroluminescence from the AlGaN nanorod LEDs was demonstrated. This method provides a novel way to grow nanorods without a metal catalyst or crystalline bulk. Furthermore, the growth of GaN nanorods on graphene buffer offers possibilities for the achievement of flexible optoelectronic devices.

## Figures and Tables

**Table 1:** The gas flow rate of precursors and dopants for all the MOCVD processes.

Step	NH_3_ (sccm)	TMGa (sccm)	TMAl (sccm)	SiH_4_ (sccm)	Cp_2_Mg (sccm)
Nitridation	1000	/	/	/	/
n-AlGaN	1000	17.5	30	500	/
nucleation islands	15	35	290	500	/
n-AlGaN nanorods	15	35	250	/	/
u-AlGaN MQWs	15	35	290	/	/
p-AlGaN	15	30	250	/	150

## References

[1] Zubia D., Hersee S.D. (1999). Nanoheteroepitaxy: The Application of nanostructuring and substrate compliance to the heteroepitaxy of mismatched semiconductor materials. J. Appl. Phys..

[2] Li S., Waag A. (2012). GaN-based nanorods for solid state lighting. J. Appl. Phys..

[3] Waag A., Wang X., Fündling S., Ledig J., Erenburg M., Neumann R., Al Suleiman M., Merzsch S., Wei J., Li S. (2011). The nanorod approach: GaN nanoleds for solid state lighting. Phys. Status Solidi C.

[4] Djavid M., Mi Z. (2016). Enhancing the light extraction efficiency of AlGaN deep ultraviolet light emitting diodes by using nanowire structures. Appl. Phys. Lett..

[5] Coulon P.M., Kusch G., Le Boulbar E.D., Chausse P., Bryce C., Martin R.W., Shields P.A. (2018). Hybrid top-down/bottom-up fabrication of regular arrays of AlN nanorods for deep-UV core-shell leds. Phys. Status Solidi B.

[6] Coulon P.M., Kusch G., Martin R.W., Shields P.A. (2018). Deep UV emission from highly ordered AlGaN/AlN core-shell nanorods. ACS Appl. Mater. Interfaces.

[7] Zhuang Z., Guo X., Zhang G., Liu B., Zhang R., Zhi T., Tao T., Ge H., Ren F., Xie Z. (2013). Large-scale fabrication and luminescence properties of GaN nanostructures by a soft UV-curing nanoimprint lithography. Nanotechnology.

[8] Wu S., Wang L., Yi X., Liu Z., Wei T., Yuan G., Wang J., Li J. (2017). Influence of lateral growth on the optical properties of GaN nanowires grown by hydride vapor phase epitaxy. J. Appl. Phys..

[9] Wu S., Wang L., Yi X., Liu Z., Yan J., Yuan G., Wei T., Wang J., Li J. (2018). Crystallographic orientation control and optical properties of GaN nanowires. RSC Adv..

[10] Li Y., Zhao Y., Wei T., Liu Z., Duan R., Wang Y., Zhang X., Wu Q., Yan J., Yi X. (2017). Van Der Waals epitaxy of GaN-based light emitting diodes on wet-transferred multilayer graphene film. Jpn. J. Appl. Phys..

[11] Mazid Munshi A., Weman H. (2013). Advances in semiconductor nanowire growth on graphene. Phys. Status Solidi Rapid Res. Lett..

[12] Liu X., Wang F., Wu H., Wang W. (2014). Strengthening metal nanolaminates under shock compression through dual effect of strong and weak graphene interface. Appl. Phys. Lett..

[13] Kumaresan V., Largeau L., Madouri A., Glas F., Zhang H., Oehler F., Cavanna A., Babichev A., Travers L., Gogneau N. (2016). Epitaxy of GaN nanowires on graphene. Nano Lett..

[14] Chung K., Yoo H., Hyun J.K., Oh H., Tchoe Y., Lee K., Baek H., Kim M., Yi G.C. (2016). Flexible GaN light emitting diodes using GaN microdisks epitaxial laterally overgrown on graphene dots. Adv. Mater..

[15] Heilmann M., Munshi A.M., Sarau G., Gobelt M., Tessarek C., Fauske V.T., van Helvoort A.T., Yang J., Latzel M., Hoffmann B. (2016). Vertically oriented growth of GaN nanorods on Si using graphene as an atomically thin buffer layer. Nano Lett..

[16] Zeng Q., Chen Z., Zhao Y., Wei T., Chen X., Zhang Y., Yuan G., Li J. (2016). Graphene-assisted growth of high-quality AlN by metalorganic chemical vapor deposition. Jpn. J. Appl. Phys..

[17] Qi Y., Wang Y., Pang Z., Dou Z., Wei T., Gao P., Zhang S., Xu X., Chang Z., Deng B. (2018). Fast Growth of strain-free AlN on graphene-buffered sapphire. J. Am. Chem. Soc..

[18] Gupta P., Rahman A.A., Hatui N., Gokhale M.R., Deshmukh M.M., Bhattacharya A. (2013). MOVPE growth of semipolar III-nitride semiconductors on CVD graphene. J. Cryst. Growth.

[19] Lin Y.T., Yeh T.W., Dapkus P.D. (2012). Mechanism of selective area growth of GaN nanorods by pulsed mode metalorganic chemical vapor deposition. Nanotechnology.

[20] Lin Y.T., Yeh T.W., Nakajima Y., Dapkus P.D. (2014). Catalyst-free GaN nanorods synthesized by selective area growth. Adv. Funct. Mater..

[21] Journot T., Bouchiat V., Gayral B., Dijon J., Hyot B. (2018). Self-assembled UV photodetector made by direct epitaxial GaN growth on graphene. ACS Appl. Mater. Interfaces.

[22] Calizo I., Bejenari I., Rahman M., Liu G., Balandin A.A. (2009). Ultraviolet Raman microscopy of single and multilayer graphene. J. Appl. Phys..

[23] Sarau G., Lahiri B., Banzer P., Gupta P., Bhattacharya A., Vollmer F., Christiansen S. (2013). Enhanced Raman scattering of graphene using arrays of split ring resonators. Adv. Opt. Mater..

[24] Zafar Z., Ni Z.H., Wu X., Shi Z.X., Nan H.Y., Bai J., Sun L.T. (2013). Evolution of Raman spectra in nitrogen doped graphene. Carbon.

[25] Chae S.J., Kim Y.H., Seo T.H., Duong D.L., Lee S.M., Park M.H., Kim E.S., Bae J.J., Lee S.Y., Jeong H. (2015). Direct growth of etch pit-free GaN crystals on few-layer graphene. RSC Adv..

[26] Reshchikov M.A., Morkoç H. (2005). Luminescence properties of defects in GaN. J. Appl. Phys..

[27] Watanabe S., Yamada N., Nagashima M., Ueki Y., Sasaki C., Yamada Y., Taguchi T., Tadatomo K., Okagawa H., Kudo H. (2003). Internal quantum efficiency of highly-efficient In_x_Ga_1−x_N-based near-ultraviolet light emitting diodes. Appl. Phys. Lett..

[28] Mickevičius J., Jurkevičius J., Kadys A., Tamulaitis G., Shur M., Shatalov M., Yang J., Gaska R. (2015). Low-temperature redistribution of non-thermalized carriers and its effect on efficiency droop in AlGaN epilayers. J. Phys. D Appl. Phys..

[29] Choi J.K., Huh J.H., Kim S.D., Moon D., Yoon D., Joo K., Kwak J., Chu J.H., Kim S.Y., Park K. (2012). One-step graphene coating of heteroepitaxial GaN films. Nanotechnology.

[30] Liu X., Le B.H., Woo S.Y., Zhao S., Pofelski A., Botton G.A., Mi Z. (2017). Selective area epitaxy of AlGaN nanowire arrays across nearly the entire compositional range for deep ultraviolet photonics. Opt. Express.

